# Deconvolution at the single-cell level reveals ovarian cell-type-specific transcriptomic changes in PCOS

**DOI:** 10.1186/s12958-024-01195-w

**Published:** 2024-02-19

**Authors:** Shumin Li, Yimeng Li, Yu Sun, Gengchen Feng, Ziyi Yang, Xueqi Yan, Xueying Gao, Yonghui Jiang, Yanzhi Du, Shigang Zhao, Han Zhao, Zi-Jiang Chen

**Affiliations:** 1 Department of Reproductive Medicine, Ren Ji Hospital, Shanghai Jiao Tong University School of Medicine, Shanghai, People’s Republic of China; 2 State Key Laboratory of Reproductive Medicine and Offspring Health, Shandong University, Jinan, Shandong People’s Republic of China; 3 National Research Center for Assisted Reproductive Technology and Reproductive Genetics, Shandong University, Jinan, Shandong People’s Republic of China; 4 Key Laboratory of Reproductive Endocrinology (Shandong University), Ministry of Education, Jinan, Shandong People’s Republic of China; 5 Research Unit of Gametogenesis and Health of ART-Offspring, Chinese Academy of Medical Sciences (No.2021RU001), Jinan, Shandong People’s Republic of China; 6 Shandong Key Laboratory of Reproductive Medicine, Shandong Provincial Hospital Affiliated to Shandong First Medical University, Jinan, Shandong People’s Republic of China; 7 Shanghai Key Laboratory for Assisted Reproduction and Reproductive Genetics, Shanghai, People’s Republic of China; 8 Department of Obstetrics and Gynecology, Qilu Hospital of Shandong University, Jinan, Shandong People’s Republic of China; 9 Gusu School, Nanjing Medical University, Nanjing, Jiangsu People’s Republic of China

**Keywords:** Polycystic ovary syndrome, Deconvolution, Granulosa cells, scRNA-seq, Bulk RNA-seq

## Abstract

**Background:**

Polycystic ovary syndrome (PCOS) is one of the most common reproductive endocrine disorders in females of childbearing age. Various types of ovarian cells work together to maintain normal reproductive function, whose discordance often takes part in the development and progression of PCOS. Understanding the cellular heterogeneity and compositions of ovarian cells would provide insight into PCOS pathogenesis, but are, however, not well understood. Transcriptomic characterization of cells isolated from PCOS cases have been assessed using bulk RNA-seq but cells isolated contain a mixture of many ovarian cell types.

**Methods:**

Here we utilized the reference scRNA-seq data from human adult ovaries to deconvolute and estimate cell proportions and dysfunction of ovarian cells in PCOS, by integrating various granulosa cells(GCs) transcriptomic data.

**Results:**

We successfully defined 22 distinct cell clusters of human ovarian cells. Then after transcriptome integration, we obtained a gene expression matrix with 13,904 genes within 30 samples (15 control vs. 15 PCOS). Subsequent deconvolution analysis revealed decreased proportion of small antral GCs and increased proportion of *KRT8*^high^ mural GCs, *HTRA1*^high^ cumulus cells in PCOS, especially increased differentiation from small antral GCs to *KRT8*^high^ mural GCs. For theca cells, the abundance of internal theca cells (TCs) and external TCs was both increased. Less *TCF21*^high^ stroma cells (SCs) and more *STAR*^high^ SCs were observed. The proportions of NK cells and monocytes were decreased, and T cells occupied more in PCOS and communicated stronger with inTCs and exTCs. In the end, we predicted the candidate drugs which could be used to correct the proportion of ovarian cells in patients with PCOS.

**Conclusions:**

Taken together, this study provides insights into the molecular alterations and cellular compositions in PCOS ovarian tissue. The findings might contribute to our understanding of PCOS pathophysiology and offer resource for PCOS basic research.

**Supplementary Information:**

The online version contains supplementary material available at 10.1186/s12958-024-01195-w.

## Introduction

Polycystic ovary syndrome (PCOS) is one of the most common disorders in women of reproductive age, with a global prevalence of up to 15% [1]. PCOS is characterized by a series of interrelated reproductive abnormalities, including disturbances in luteinizing hormone (LH) and follicle-stimulating hormone (FSH) secretion, increased androgen production, chronic anovulation and polycystic ovarian morphology [2]. Follicular developmental disorder is one of the most common symptoms of PCOS, as the main cause of anovulation and infertility.

Follicle recruitment, growth and ovulation as well as the acquisition of oocyte developmental competence in the ovary require the participation of a series of ovarian cells [3–5], including regulation of ovarian extracellular matrix (ECM) formation, vasculature, signal transduction and steroidogenesis [6, 7]. Arrest of antral follicle growth in PCOS is associated with an abnormal endocrine environment involving hypersecretion of luteinizing hormone (LH), insulin, hyperandrogenism and dyscoordination of various ovarian cells [6]. An intrinsic abnormality in PCOS can affect the very earliest, gonadotrophin independent, stages of follicle development and normal function of ovarian cells [6, 8]. Previous studies were undertaken to elucidate the characteristics of cells isolated from PCOS patients [9–12]. However, the cells isolated were not pure enough with many other types of cells mixed. The cellular composition and heterogeneity of ovarian cells in PCOS still remain unclear.

In this study, we used the transcriptome datasets of PCOS cases GCs, combined with scRNA-seq data of human adult ovarian tissues [5], to delve into the transcriptome characteristics of ovarian cells from PCOS cases. We identified the cellular composition and molecular alterations in ovarian cells from controls and PCOS at single-cell resolution. The results of the study provide a theoretical basis for the understanding of PCOS pathogenesis.

## Methods

### Ethics statement

The datasets used in this study were taken from previously published anonymized public resources and therefore did not require additional approval from the institutional research ethics committee.

### Bulk RNA-seq analysis

After downloading the expression matrix from GEO database, we integrated the expression matrix together and corrected the batch effect of each study by using *Combat* function in sva R package. Principal component analysis (PCA) was performed using PCAtools R package to compare the correction effects for batch effect. After normalization, we used limma R package for differential expression analysis and differentially expressed genes (DEGs) were identified by |log2Fold Change| >1 and p-value < 0.05.

### Single cell RNA-seq analysis

Seurat 4.3 was used to preprocess the matrix downloaded and processing steps were based on the code uploaded in that study. Cell filtering was conducted according to the following parameters: the total number of expressed genes/cell was 200 < nCount < 2500; the total number of UMIs/cell was 300 < nFeature < 15,000; genes mapping to mitochondrial genes < 10%. Cells with more than 6% of UMIs mapping to dissociation-induced genes was also removed in the subsequent analysis. *NormalizeData* function was used for normalization. Cell type annotation was done manually. Differential expression analysis was performed via *FindAllMarkers* function (min.pct = 0.25, thresh.use = 0.25) to identify the marker genes in the cluster compared with all the rest clusters.

### Pathway enrichment

Gene Set Enrichment Analysis (GSEA) was performed using WebGestalt (www.webgestalt.org/), and functional database was Reactome. Pathway enrichment for DEGs and marker genes was performed using Metascape (https://metascape.org/). Bubble plot and volcano plot were visualized using ggplot2 R package.

### Impute cell compositions with CIBERSORT

Deconvolution was conducted by CIBERSORT R package. All marker genes identified in each cluster were integrated together as the signature matrix. We set permutations to 1000. Samples with *P* < 0.05 were included for further study.

### Pseudotime analysis of different GC clusters

Single-cell trajectory and pseudotime analyses were conducted using Monocle3 R package. Significant switching genes were identified using GeneSwitches R package. Binarize_cutoff was set to 0.2 in *binarize_exp* function. Genes that varied over the trajectory were analyzed using graph-autocorrelation. The co-regulated genes were then grouped into fifteen modules via Louvain community analysis with a set resolution of 0.0001.

### Cell-to-cell communication analyses

Cell-to-cell communication analyses were conducted using Cellchat R package (v1.6.1) to infer the interactions between TCs, SCs and immune cells.

### Connectivity map (CMap) analysis

Query tool (https://clue.io/query) in CLUE platform in Connectivity map (CMap) website was used. The query parameters were set as below: Gene expression (L1000), Touchstone, Individual query and 1.0 version. Upregulated and downregulated DEGs which were also the top markers of eight altered cell clusters were uploaded separately in the two different boxes.

### Statistical analyses

R 4.30 was used to perform statistical analyses. Comparison between two groups used Student’s test. *P* < 0.05 was considered statistically significant with a signal * and not significant without any marking.

### Data sources

**Fig. 1:**
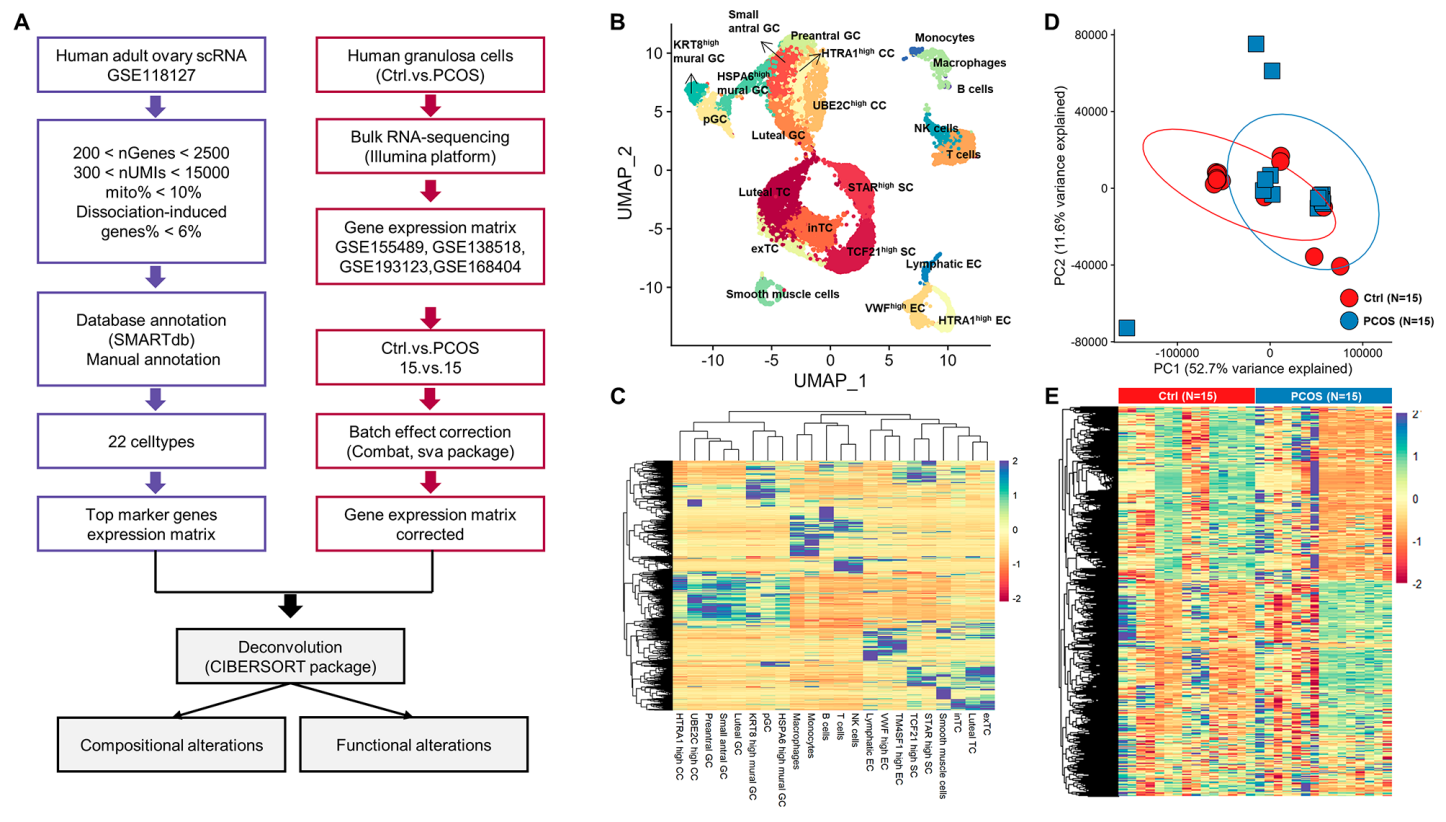
scRNA sequencing and bulk-RNA sequencing data analysis. **A** Flowchart of data processing in the study. **B** UMAP cluster map revealing 22 specific clusters representing the major ovarian cell types in human adult ovaries. **C** Heatmap of marker genes of each cluster from 22 specific cell clusters. **D** PCA plot of granulosa cells transcriptome data from 15 controls and 15 PCOS patients. **E** Heatmap of expression of 13,904 genes all detected in these 30 samples

Transcriptomic data from all granulosa cell samples performed through Illumina platform was obtained from the GEO database, including GSE155489, GSE138518, GSE193123 and GSE168404. PCOS patients diagnosed in these studies were referred to Rotterdam criteria [13]. scRNA sequencing data from the GSE118127 cohort [5], which consists of single-cell sequencing data from 36 human adult normal ovarian tissues. The entire workflow was shown in Fig. 1.

## Results

### Single-cell RNA sequencing and bulk-RNA sequencing data analysis

We utilized the scRNA-seq raw data from 36 human adult normal ovarian tissues in the GSE118127 cohort (Fig. 1A). After quality control and removing dissociation effects and ribosome genes, 15,614 cells containing 19,893 genes were included. Through comprehensive analysis using tools such as Clustertree (Supplementary Fig. 1) and extensive literature review, we have successfully defined 22 distinct cell clusters of human ovarian cells. They are progenitor granulosa cells (pGC), preantral granulosa cells, small antral granulosa cells, *HSPA6*^high^ mural granulosa cells, *KRT8*^high^ mural granulosa cells, *UBE2C*^high^ cumulus cells, *HTRA1*^high^ cumulus cells, luteal granulosa cells, internal theca cells (inTC), external theca cells (exTC), luteal theca cells, *TCF21*^high^ stroma cells, *STAR*^high^ stroma cells, *VWF*^high^ endothelial cells, *TM4SF1*^high^ endothelial cells, lymphatic endothelial cells, smooth muscle cells (SMC), T cells, B cells, NK cells, macrophages and monocytes (Fig. 1B). By *FindAllMarker* function (log2Fold change > 0.25, top 100), we obtained a gene matrix of each cell type with total 1363 top marker genes as the deconvolution reference (Fig. 1C) (Supplementary Table 1).

Subsequently, we obtained publicly available PCOS GCs transcriptomic sequencing data from the GEO database. A total of 30 RNA-seq datasets, including 15 PCOS and 15 control, from four different studies [9–12] were included for further analysis (Fig. 1D). After data integration and batch correction, a total of 13,904 genes were all detected in these 30 samples for further deconvolution using CIBERSORT (Fig. 1E) (Supplementary Table 2).

After integration, we firstly performed Gene Set Enrichment Analysis (GSEA) and differential genes enrichment analysis with the integrated gene expression matrix. We identified a total of 1345 differentially expressed genes (DEGs) using the *limma* software package, with 261 upregulated DEGs and 1084 downregulated DEGs (Supplementary Table 2). Both the enrichment results revealed that there were similar enhanced pathways including protein digestion and absorption and ECM-receptor interaction, while there was also concordant suppression of immune related pathway, such as cytokine-cytokine receptor interaction and chemokine signaling pathway in PCOS (Supplementary Fig. 2). In other words, the global and differential expression pattern of transcriptome between PCOS and control demonstrated the similar molecular signatures compared to preliminary studies, indicating the reliability and usability of our integrated data.

### Changes of the ovarian cell compositions in PCOS

**Fig. 2:**
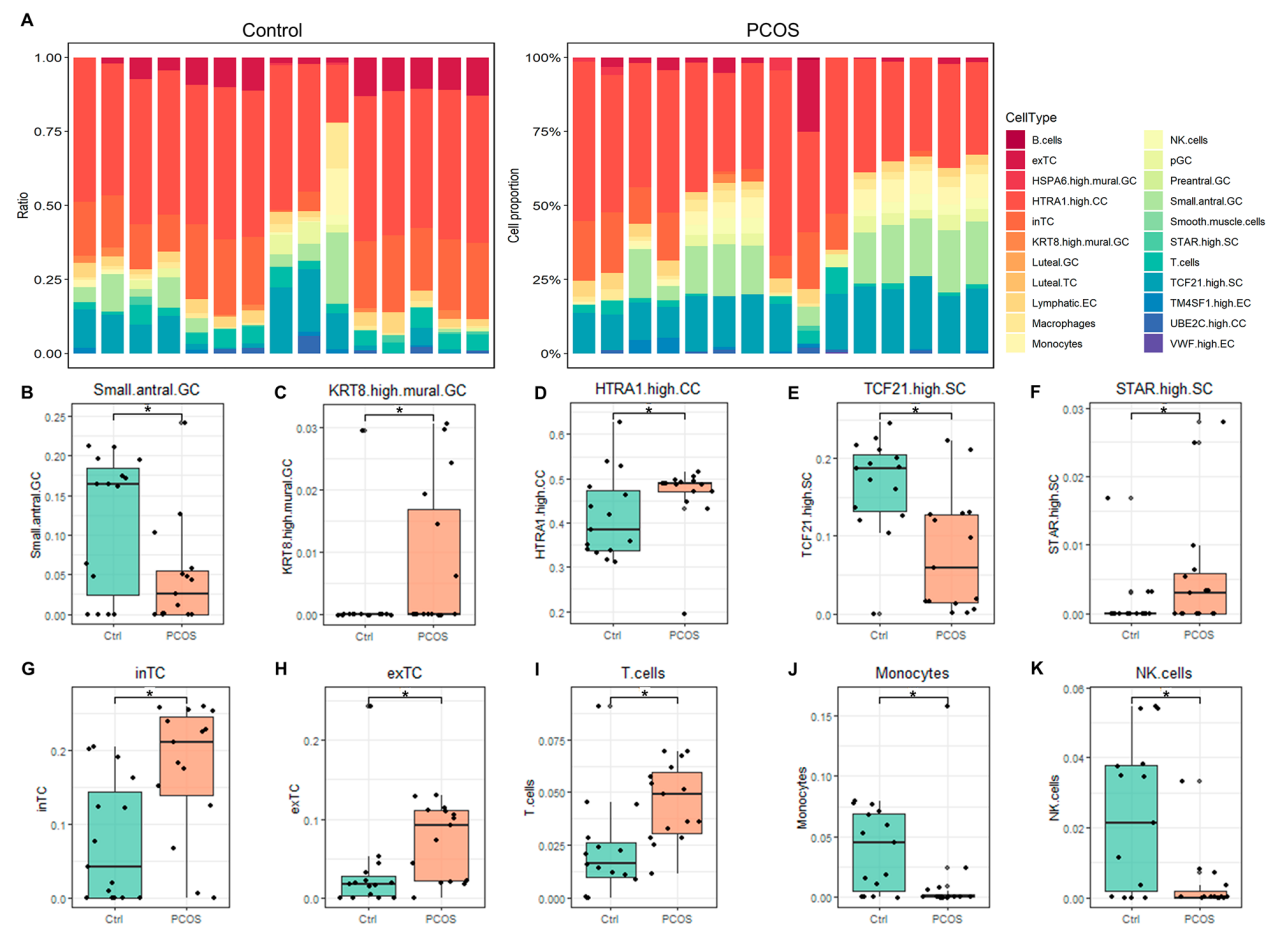
Changes of the ovarian cell compositions in PCOS. **A** Column stacked plots of the proportions of 22 cell clusters in each sample in control and PCOS groups. (**B**-**K**) Boxplots of clusters with altered proportions between two groups, including small antral GCs, *KRT8*^high^ mural GCs, *HTRA1*^high^ CC, *TCF21*^high^ SC, *STAR*^high^ SC, inTC, exTC, T cells, monocytes and NK cells. *Student’s* t test. *P* < 0.05 was considered statistically significant with a signal * and not significant without any marking

To describe the altered cell compositions in women with and without PCOS, we deconvolved 30 samples bulk-RNA sequencing data according to the maker genes matrix of the 22 cell clusters. We found the highest percentage of cells in the sample was *HTRA1*^high^ cumulus cells (CC), followed by inTC, *TCF21*^high^ SC, small antral GC and exTC etc. (Fig. 2A, Supplementary Table 3). Only three clusters in all 22 clusters of ovarian cells were not detected in all 30 samples (Supplementary Table 3). This result suggested the cells isolated from the patients were not pure enough and were mixed with a variety of other cell types.

In order to explore the difference of cellular compositions between PCOS and control, we performed *Student’s* t test and found the decreased proportion of small antral GCs but increased population of *KRT8*^high^ mural GC and *HTRA1*^high^ CC (Fig. 2B and D). Moreover, we observed the much less *TCF21*^high^ SC, but more *STAR*^high^ SC, inTC and exTC in PCOS (Fig. 2E and H). We also found the altered compositions of immune cells, including more T cells but less monocytes and NK cells in PCOS (Fig. 2I and K).

### GCs dysfunction along with the disrupted GCs differentiation in PCOS

**Fig. 3:**
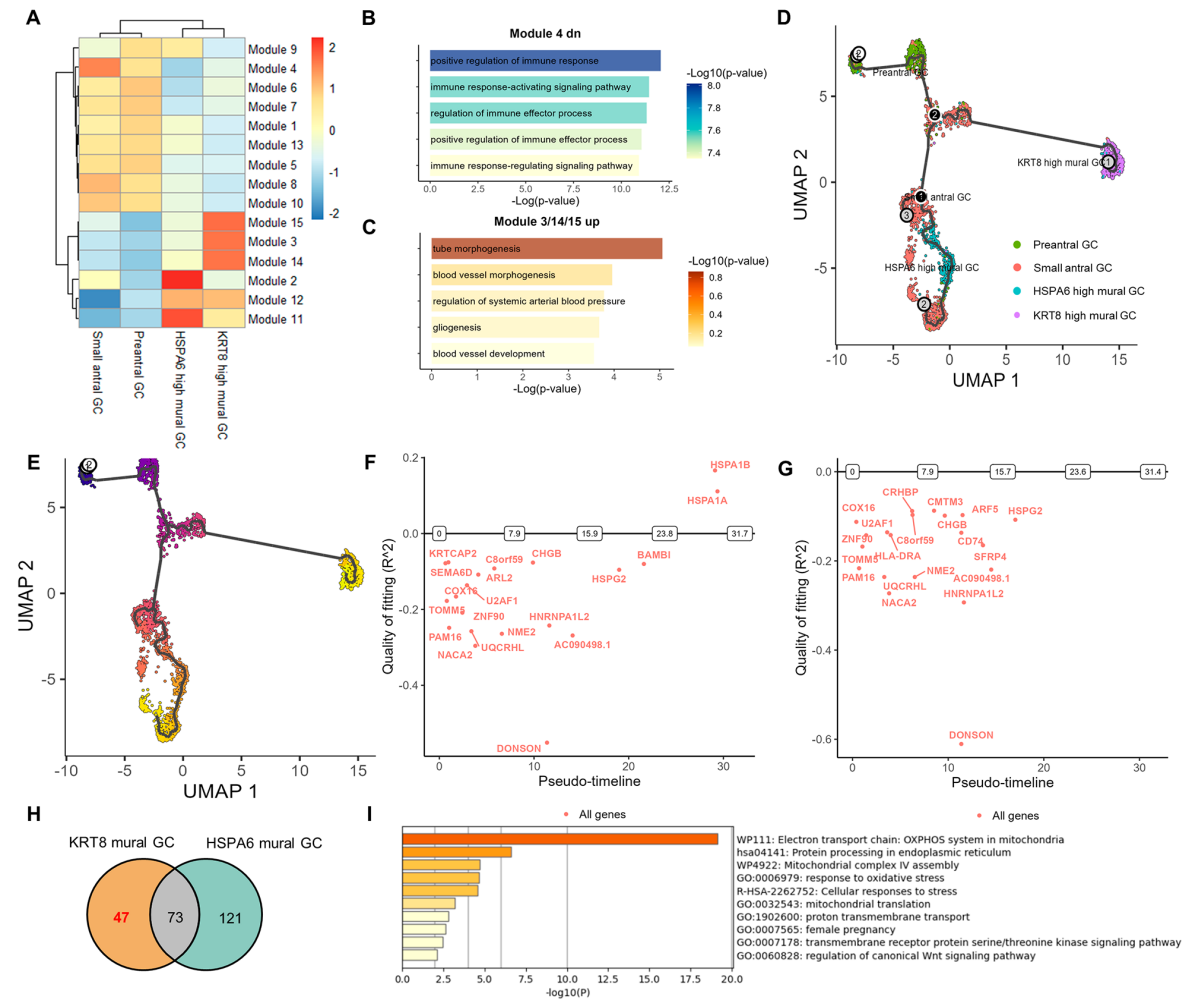
GCs dysfunction along with the disrupted GCs differentiation in PCOS. **A** Heatmap showing the core gene in modules regulating GCs differentiation in Monocle 3 (resolution = 0.0001). **B** The top 5 enriched GO (biological pathway) terms of downregulated DEGs in module 4. **C** The top 5 enriched GO (biological pathway) terms of upregulated DEGs in module 3/14/15. **D** Monocle 3 generated pseudotemporal trajectory of preantral GCs, small antral GCs, *KRT8*^high^ mural GCs and *HSPA6*^high^ mural GCs. **E** The granulosa cell trajectory predicted by Monocle 3 and visualized by UMAP. Cells were ordered in pseudotime colored in a gradient from purple to yellow. **F** Top 15 significant switching genes were ordered in pseudotime in the trajectory, from preantral GCs, small antral GCs to *KRT8*^high^ mural GCs. **G** Top 15 significant switching genes were ordered in pseudotime in the trajectory, from preantral GCs, small antral GCs to *HSPA6*^high^ mural GCs. **H** Venn plot of all the significant switching genes in above two trajectories. **I** Pathway enrichment of the 47 significant switching genes specific in *KRT8*^high^ mural GCs differentiation trajectory

Given that GCs were the most abundant population in ovarian microenvironment, we firstly focused on the GCs characteristics in PCOS. To further elucidate the overall functions across these four different GCs clusters, we identified fifteen gene modules that underwent significant alterations (Fig. 3A, Supplementary Table 4). Module 4 was prominent in small antral GCs, the proportion of which was decreased in PCOS. We investigated the downregulated DEGs in module 4, and found that they were involved in positive regulation of immune response, indicating the diminished immune response in small antral GCs from PCOS (Fig. 3B). In the meanwhile, *KRT8*^high^ mural GC cluster was significantly increased in PCOS and modules 3, 14 and 15 were elevated in this cluster of cells. The upregulated DEGs in modules 3, 14 and 15 mainly undertook the role for tube morphogenesis (Fig. 3C). The increased *KRT8*^high^ mural GC cluster probably mediated the blood vessel morphogenesis around follicles in PCOS.

Due to the intense differentiation that occurs in granulosa cells during follicle development and significant altered abundance in *KRT8*^high^ mural GCs and small antral GCs, we then paid attention to the differentiation trajectory of preantral GCs, small antral GCs and different mural GCs by using Monocle 3 to perform a developmental trajectory analysis. Our analysis revealed preantral GCs underwent two distinct developmental trajectories, towards small antral GCs/*HSPA6*^high^ mural GCs or to *KRT8*^high^ mural GCs (Fig. 3D and E). To explore the key genes involved in cell differentiation, we then utilized *GeneSwitches* and found a series of different genes in these two developmental trajectories (Fig. 3F and G). We compared the obvious changed genes during these two trajectories (Fig. 3H), and the comparison revealed the presence of 47 genes specific in *KRT8*^high^ mural GCs developmental trajectory. According to Metascape resource website, these 47 genes (Supplementary Table 5) were suggestive to have critical functions in electron transport chain in mitochondria, protein processing in endoplasmic reticulum, cellular response to stress as well as female pregnancy (Fig. 3I). These findings suggested that the gene expression and molecular function involved in the differentiation progress to *KRT8*^high^ mural GCs were disturbed in PCOS (Fig. 3I).

### Enhanced cell communications from TCs in PCOS

**Fig. 4:**
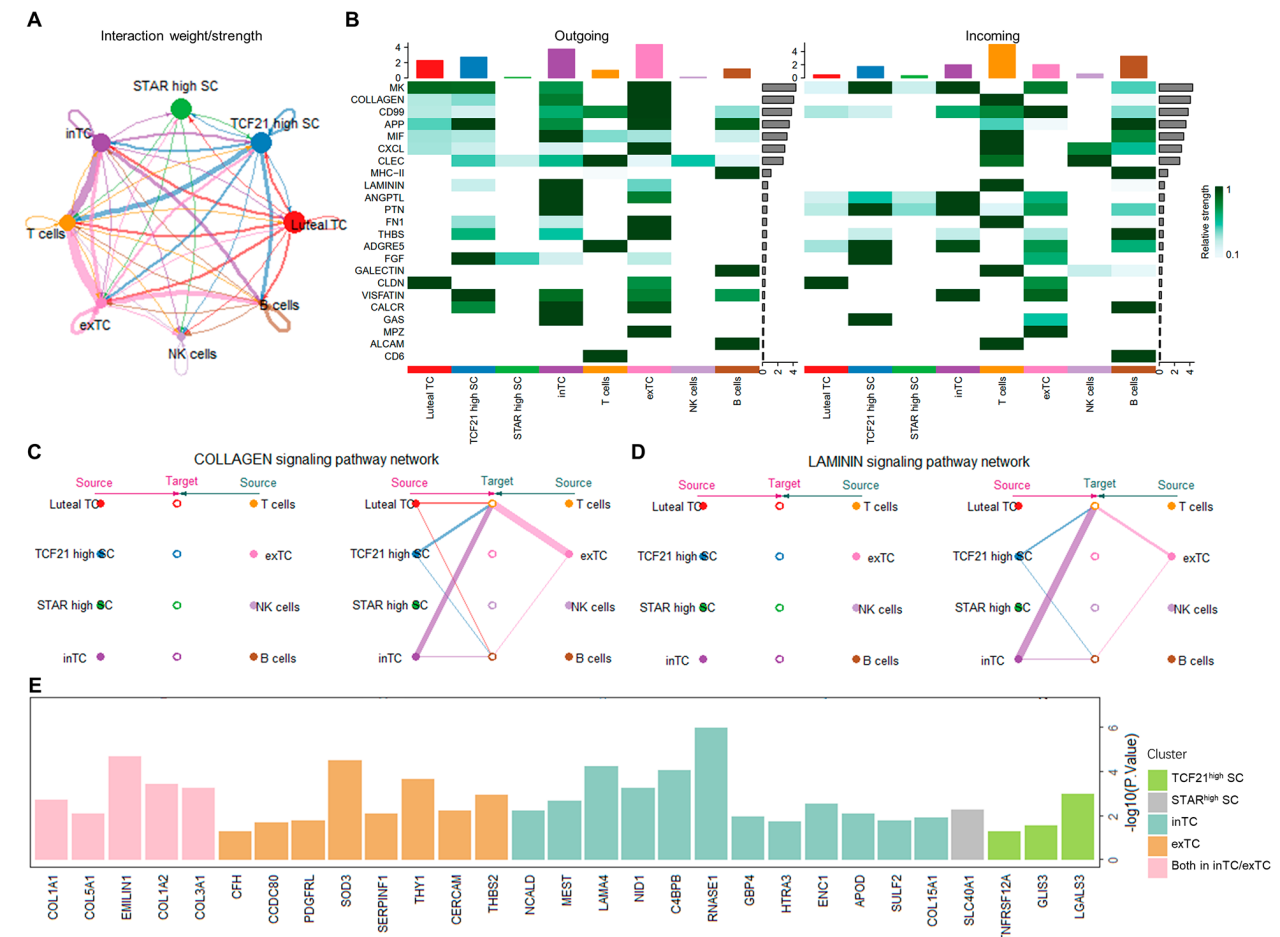
Enhanced cell communications from TCs in PCOS. **A** Network displaying the number of cell-to-cell interactions in the TC, SC and immune cell clusters. **B** Heatmap of outgoing/incoming cell-to-cell interaction signaling pathways in the TC, SC and immune cell clusters. **C** Chord plot of COLLAGEN pathway among TC, SC and immune cell clusters. **D** Chord plot of LAMININ pathway among TC, SC and immune cell clusters. **E** The bar diagram showing the intersection of the DEGs between PCOS and control groups with the TC and SC clusters marker genes, with -log10(P-value) in vertical coordinates

The ovarian stroma was mainly composed of TCs, stroma cells, immune cells and so on [14]. Considering the presence of a basal lamina between their granulosa and theca cell compartments as a blood-follicle barrier, we next aimed to investigate the cellular interactions between TCs, stroma cells and immune cells. We performed cell-to-cell communications by *Cellchat*, and the result showed that the most intense interactions between these cells were inTCs and exTCs (outgoing) and T cells (ingoing) (Fig. 4A).

To further explore the signaling between these cells, we found several signaling pathways with high intensity outgoing from inTCs and exTCs to T cells, including LAMININ and COLLAGEN (Fig. 4B). We found that inTCs and exTCs via secreting collagen and laminin had strong communication with T cells (Fig. 4C and D). Consistently, some top markers in inTCs and exTCs were the components of above pathways (Fig. 4E). These genes were determined significantly upregulated in PCOS as well, like *COL1A1*, *COL1A2* and *LAMA4* (Fig. 4C). Collectively, increased proportion of inTCs and exTCs as well as the activated LAMININ and COLLAGEN pathways in PCOS probably promoted the close communications between TCs and T cells, which could partly explain the significant increase in T cell proportion in PCOS.

### Drug candidates for improving altered cellular compositions in PCOS

**Fig. 5:**
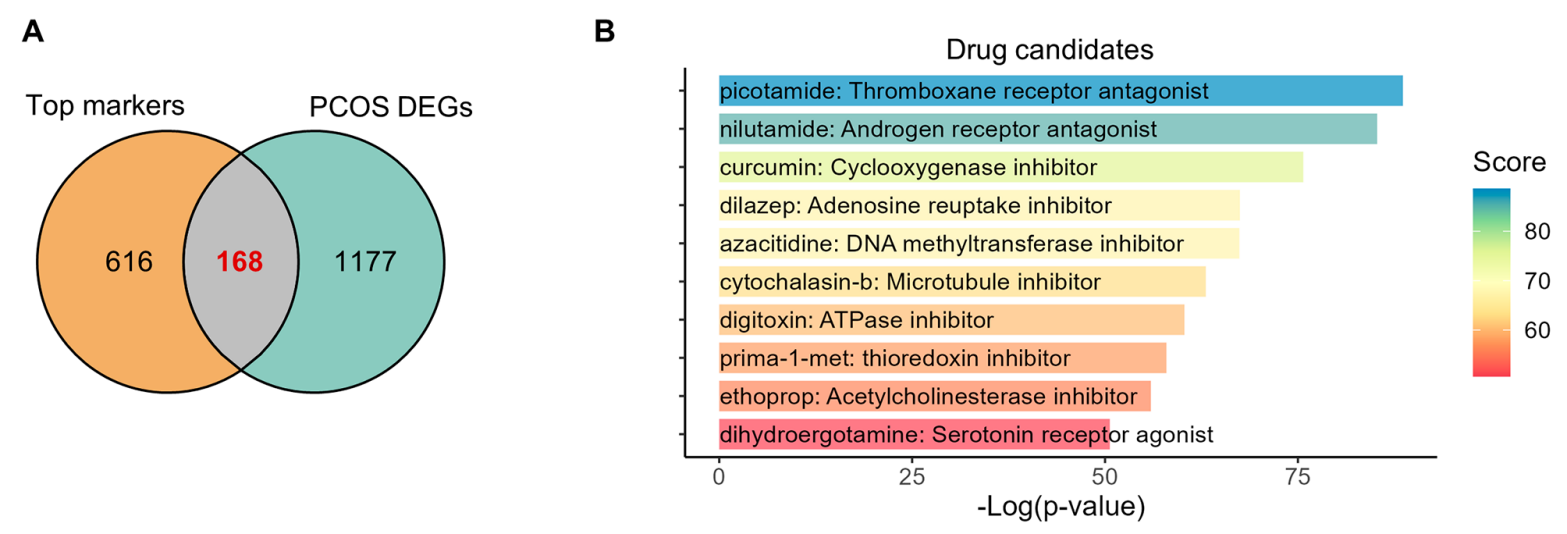
Drug candidates for improving altered cellular compositions in PCOS. **A** Venn plot of top markers of cell clusters whose proportions were altered and DEGs between PCOS and control. **B** Barplot of the top 10 predicted compounds targeting the altered cellular compositions in PCOS

Finally, aiming to tap into more therapeutic agents and targets for improving altered cellular compositions in PCOS, we decided to explore the drug candidates according to the DEGs as top markers in these altered cell clusters. The eight altered cell clusters owned 784 top markers, and among them, there were 168 DEGs between PCOS and control (Fig. 5A). We uploaded these altered secreted proteins to CMap (https://clue.io/) and retrieved a list of compounds which could be the new drugs for potential PCOS therapies (Supplementary Table 6). The top 10 predicted drug candidates were shown in Fig. 5B. The top components predicted was picotamide, which was the thromboxane receptor antagonist (Fig. 5B). Moreover, nilutamide as androgen receptor antagonist ranked second (Fig. 5B). Hence, more possible treatment strategies for PCOS were identified based on our analysis, possibly targeting to correct the disturbed cellular compositions.

## Discussion

PCOS is a polygenic and polyfactorial reproductive and endocrine disorder, affecting women of reproductive age. The etiology and pathogenesis of PCOS are diverse, including insulin resistance, hypothalamus–pituitary–ovary (HPO) axis disturbance and ovarian microenvironment disorder, etc. Moreover, the chronic inflammatory and insulin resistant state also influenced the ovarian steroidogenesis and oocyte maturation. In this study, we employed transcriptomic data from cells isolated from PCOS cases, combined with single-cell sequencing data from the ovaries, to investigate the molecular and cellular biology of ovarian tissues in women with and without PCOS. Using integration of transcriptomic data, we found the putative activation in ECM-receptor interaction and suppression in cytokine-cytokine receptor interaction. We resolved the changes in the ratio of different cell types in the ovary in PCOS, and observed the increased differentiation from small antral GCs to *KRT8*^high^ mural GCs with less small antral GCs and more *KRT8*^high^ mural GCs in PCOS. These different GCs clusters were developing, dynamically changing, and there was a progressive relationship of developmental differentiation between them. We held the concept that these different clusters owned their different gene expression profiles and these changes in profiles of different cell clusters were altered in ovarian cells of women with PCOS. Moreover, both the abundance of internal theca cells (inTCs) and external theca cells (exTCs) was increased, while less *TCF21*^high^ stroma cells (SCs) and more *STAR*^high^ SCs were observed. The proportions of NK cells and monocytes were decreased, and T cells occupied more in PCOS and communicated stronger with inTCs and exTCs.

Cytokine-cytokine receptor interaction is well investigated to be essential for regulating ovarian physiology, particularly in relation to folliculogenesis and ovulation, where they contribute to creating an environment supporting follicle selection and growth [15, 16]. Previous studies have placed great importance on the immune reaction on ovulation. Immune cells were also essential for ovulation and vascular invasion of the newly forming corpus lutea [17]. The depletion of immune cells and the attenuated cytokine signaling prior to ovulation, blocked corpus luteum formation [18]. Many mediators of LH-induced signaling cascades in ovulation are associated with inflammation, leading to the postulate that ovulation is similar to an inflammatory response [19]. In our study, after integration of all transcriptomic data, we found the obvious repression in inflammation response. The deconvolution results also revealed the smaller population of NK cells and monocytes in PCOS. These findings indicate that the cytokines in ovarian microenvironment, repression of inflammatory response along with insufficient inflammatory cells are not sufficient to bring about adequate ovulation.

ECM receptor interaction is also highly required in the development of ovarian follicles [6, 20], which not only influences follicular cell structure and endocrine function, but also affects the process of ovulation [21, 22]. For instance, collagen IV, the more concentrated in the follicular basement membrane, undergoes tremendous remodeling during follicular development and ovulation, allowing the ECM to adapt to the growing follicle and rupture the follicle during ovulation [20]. In our analysis, the excessively activated ECM receptor interaction is likely to prevent the coordinated follicular development and normal ovulation in PCOS. Consistently, the disorder in ECM receptor interaction has been investigated in PCOS [20, 23], while targeting the ECM in PCOS promotes restoration of ovulation in PCOS-model animals [24, 25]. In addition to ECM receptor interaction, many upregulated DEGs are also enriched in PI3K/Akt signaling pathway, which plays a pivotal role in regulating the impact of insulin on metabolism [26] as well as in regulating cell growth and proliferation, [27, 28]. The PI3K-Akt pathway disturbance is complicated and multifaceted [26, 29, 30], which could lead to insulin resistance [26], abnormal follicle apoptosis and proliferation [31], and the formation of ovarian cysts [32].

It is generally accepted that the follicle is a functional unit consisting of an oocyte, the granulosa cell compartment and the theca cell compartment. As the GCs proliferate, they differentiate into three distinct cell populations: cumulus GCs that surround and support the oocyte, antral GCs adjacent to the follicular antrum, and mural GCs adjacent to the basal lamina that separates the GCs compartment from the TC compartment. After ovulation, mural GCs gradually transform into part of luteal GCs, which are involved in a series of subsequent hormone-regulating processes. Mural GCs produce estrogen during the follicular phase and progesterone after ovulation [33, 34].

In our study, we observed the increased abundance of *KRT8*^high^ mural GCs and those genes involved in the development were associated with mitochondria, protein processing and female pregnancy. Previously, a close association was found between changes in KRT8/KRT18 expression and cell death/cell survival events in the human GCs lineage [35]. In the previous research, large secondary and atretic early antral folliclesdisplayed low KRT8/KRT18 expression. However, early growing and some large antral follicles displayed high KRT8/KRT18 expression, in which apoptosis was scarce. Primordial follicles showing high KRT8/KRT18 levels were those predominantly recruited into the growing pool. This research demonstrated that KRT8/KRT18 could play a vital role in regulating primordial follicle growth and the increase in *KRT8*^high^ mural GCs in PCOS could partially explain high follicle count in PCOS.

TCs can produce paracrine mediators of ovulation [19], containing an inner layer of steroidogenic cells called the theca interna, an outer layer of fibroblast-like theca externa, and a rich vascular network. TCs produce steroidal and nonsteroidal factors, influencing other cells proliferation and differentiation during folliculogenesis. Moreover, TCs in growing follicles produced androgens in response to LH. It was discovered that TCs-derived androgens were then converted to estradiol by aromatase enzyme in GCs. A series of studies in vitro and in vivo elucidated the excessive TC androgen production in PCOS [36–38]. Based on the deconvolution results in our study, a marked increase in the proportion of TCs was observed in patients with PCOS [39], not only inTCs but also exTCs, thereby leading to the over-production of androgen.

Reportedly, localization of leukocyte subsets in the follicle wall and in the corpus luteum underwent alterations throughout the human menstrual cycle [40]. Some immune cells like T cells were present in high numbers in the collagen-rich tissues, including the thecal layer, of the follicle wall. The fibrous proteins like laminins and collagens directly or indirectly could influence the migration, phenotype, and function of T cells [41]. In our study, we found the strong communications between T cells and inTCs and exTCs, especially through COLLAGEN and LAMININ signaling pathways. The upregulated genes involved in these two pathways expressing in TCs along with the increased proportions of TCs may induce the increased proportion of T cells.

## Conclusions

In conclusion, we applied scRNA-seq data of human ovary to deconvolve the transcriptome data in PCOS patients, and discovered disturbances in cellular proportions, function, differentiation, and communications in PCOS. In the meanwhile, the molecular and cellular heterogeneity exploration can also provide the theoretical basis for PCOS basic research and potential therapies. As with the popularity of cell type decomposition methods, we suggested that using scRNA sequencing data to deeper elucidate the cellular compositions in samples of different diseases with scarce source.

### Electronic supplementary material

Below is the link to the electronic supplementary material.

Supplementary Material 1

Supplementary Material 2

## Data Availability

No datasets were generated or analysed during the current study.
