# Supplementary material for: Deconvolution at the single-cell level reveals ovarian cell-type-specific transcriptomic changes in PCOS
Source: Reprod Biol Endocrinol. 2024 Feb 19;22:24. doi: 10.1186/s12958-024-01195-w (PMC10875798; doi:10.1186/s12958-024-01195-w)
Supplement: Supplementary file 2 — Supplementary Material 2 [file 12958_2024_1195_MOESM2_ESM.docx]

**Supplementary figure**

**
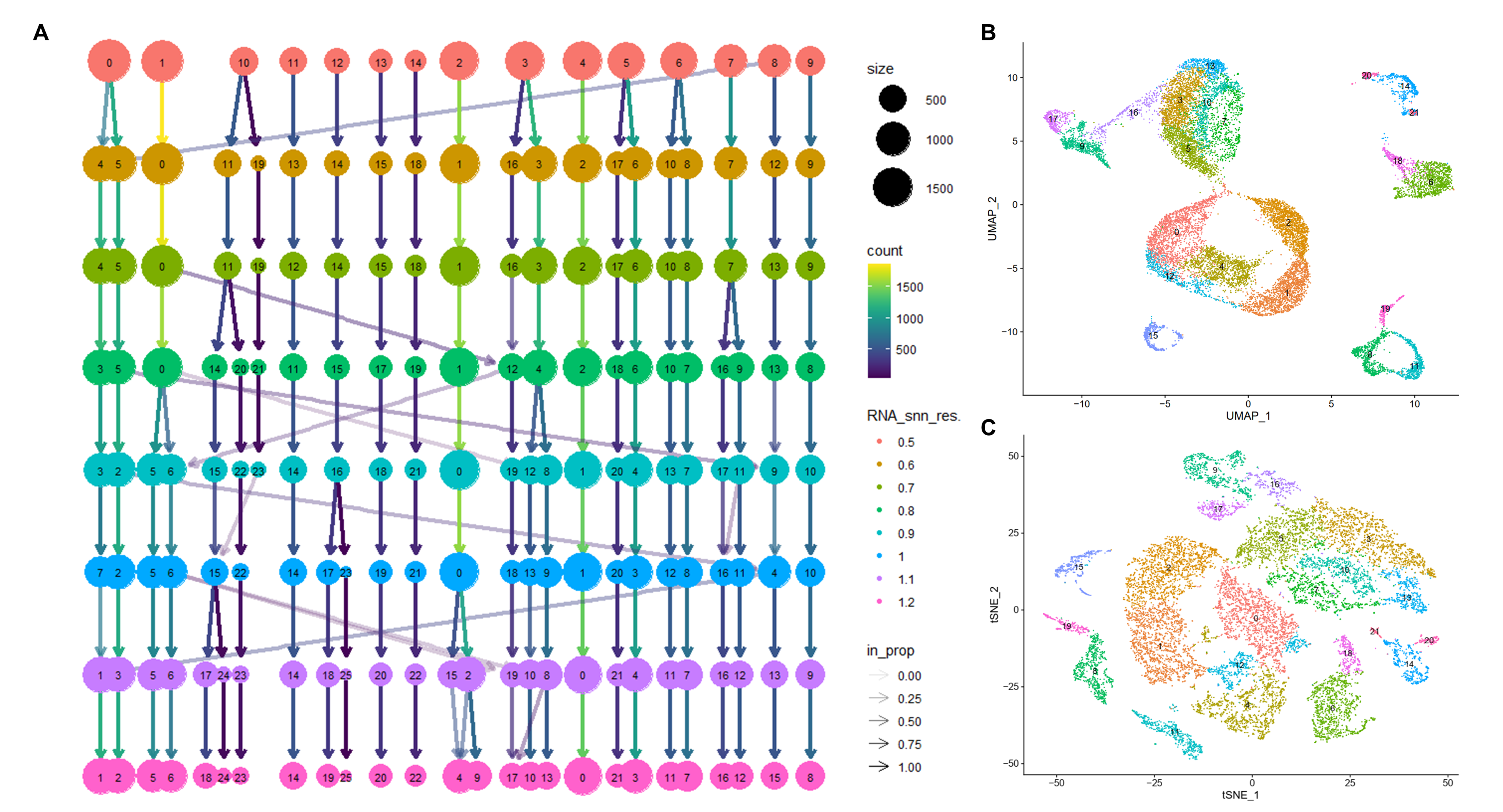
**

**Supplementary figure 1 scRNA~~Sc-RNA~~ sequencing data analysis**

1. Cluster tree of cell clusters identification in different resolution. Resolution 0.8 was chosen for further analysis.
2. UMAP plot of ovary clusters.
3. t-SNE ~~T-SNE~~ plot of ovary clusters.


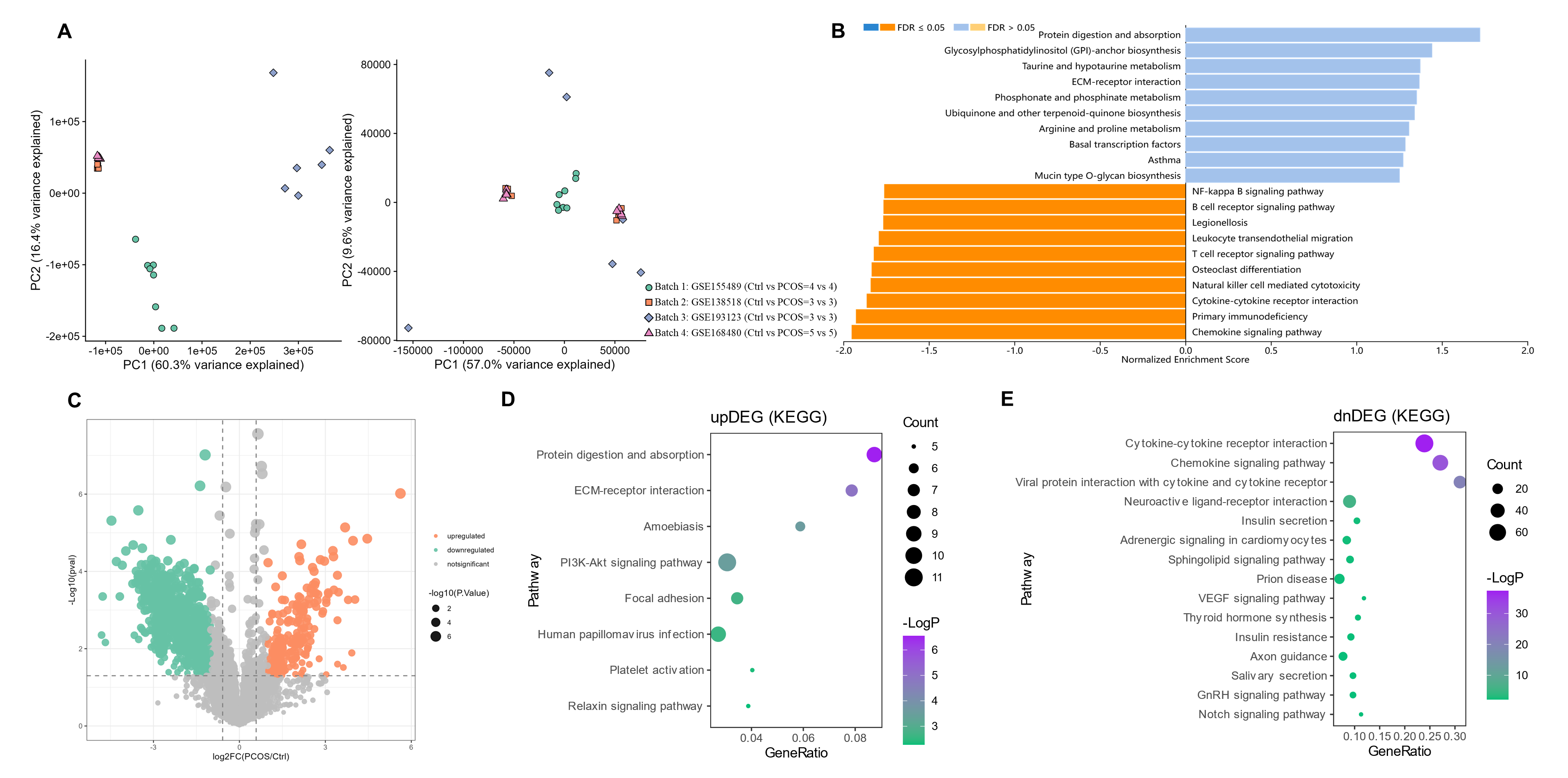


**Supplementary figure 2 Differential analysis of integrated RNA-seq between PCOS and Ctrl**

1. PCA plot of granulosa cells transcriptome data from 15 controls and 15 PCOS patients, labeled in different shapes and colors according to different batches. Left: before batch correction; Right: after batch correction. Batch 1: GSE155489 (Control vs PCOS=4 vs 4); Batch 2: GSE138518 (Control vs PCOS=3 vs 3); Batch 3: GSE193123 (Control vs PCOS=3 vs 3); Batch 4: GSE168480 (Control vs PCOS=5 vs 5)
2. Pathway enrichment of GSEA analysis between PCOS and Ctrl.
3. Volcano plot of differential expression analysis of integrated transcriptomic data. X-axis: log2(FoldChange); Y-axis -log10(p-value). DEGs (|FoldChange|>2, p-value<0.05) were labeled. Orange dots: upregulated DEGs; Green dots: downregulated DEGs.
4. KEGG pathway enrichment of upregulated DEGs.
5. KEGG pathway enrichment of downregulated DEGs.
